# Exploring New Product Development: Acceptance of Textured Vegetable Protein With Baru By‐Product in Plant‐Based Burgers

**DOI:** 10.1002/fsn3.72009

**Published:** 2026-06-08

**Authors:** Ana Paula Rebellato, Bruna dos Reis Gasparetto Cruz, Victor Guilherme Sebastião, Mirian dos Santos, Joyce Grazielle Siqueira Stürmlinger, Marise Aparecida Rodrigues Pollonio, Caroline Joy Steel

**Affiliations:** ^1^ Department of Food Engineering and Technology, School of Food Engineering Universidade Estadual de Campinas (UNICAMP) Campinas Brazil; ^2^ Department of Food Technology and Bioprocess Engineering Max Rubner‐Institute, Federal Research Institute of Nutrition and Food Karlsruhe Germany

**Keywords:** alternative proteins, Brazilian biodiversity, Cerrado, consumer acceptance, plant proteins, sustainable diets

## Abstract

The growing demand for sustainable meat alternatives has increased interest in plant‐based burgers formulated with textured vegetable proteins (TVPs). This study aimed to develop and evaluate plant‐based burgers containing TVPs enriched with defatted baru flour (DBF), a Brazilian by‐product rich in proteins, lipids, and dietary fiber. Formulations were prepared using soy protein concentrate and vital wheat gluten, including four DBF levels and a control without DBF. Formulations E1 (4% DBF) and E16 (10% DBF) showed higher protein and lipid contents compared to the control, as well as a darker color. Sensory evaluation (*n* = 87) indicated that these formulations achieved higher acceptance scores (*p* < 0.05) for appearance, texture, and overall impression compared to the control. Microbiological analyses confirmed product stability during 60 days of frozen storage. Overall, the incorporation of defatted baru flour improved the nutritional and technological properties of plant‐based burgers. These results support the potential use of baru by‐product as a functional ingredient in plant‐based formulations, contributing to the valorization of native resources from the Cerrado biome.

## Graphical abstract

Textured vegetable proteins were produced by thermoplastic extrusion using soy protein concentrate, vital wheat gluten, and defatted baru flour, a protein‐ and fiber‐rich by‐product from the Brazilian Cerrado. The resulting plant‐based burgers were evaluated for nutritional composition, technological properties, microbiological quality, and sensory acceptance. Incorporation of defatted baru flour improved nutritional quality and consumer acceptance, highlighting its potential as a sustainable functional ingredient for plant‐based meat alternatives.

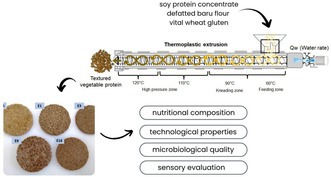

## 1 Introduction

Plant‐based proteins have attracted global interest due to their potential for developing meat analogs. These products are developed as sustainable alternatives to conventional animal production systems, aiming to mitigate environmental impacts, human health risks, and ethical issues related to animal welfare (Olegario et al. 2025; Sendhil et al. 2024). To achieve adequate consumer acceptance, analogs must mimic the sensory characteristics of conventional meat, such as appearance, texture, and flavor, while also providing equivalent nutritional profiles (McClements and Grossmann 2021a; Shaghaghian et al. 2022). However, despite advances, the market still offers relatively few textured plant proteins that combine strong technological and functional properties, representing a significant challenge for developing products with sensory and nutritional quality comparable to traditional meat (dos Reis Gasparetto Cruz et al. 2025).

The production of meat analogs, such as plant‐based burgers, involves different processing operations. Each operation must be adapted to the specific properties of plant proteins, which differ from animal proteins in solubility, denaturation temperature, and gelling capacity, thus requiring distinct processing conditions to attain physicochemical and sensory characteristics similar to those of animal protein (McClements and Grossmann 2021b). Techniques such as extrusion have therefore gained prominence for transforming globular plant proteins into fibrous or porous structures. Indeed, textured plant proteins are already used in analogs such as burgers, nuggets, and plant‐based sausages (Arora et al. 2020; Baune et al. 2022; McClements and Grossmann 2021b).

Thermoplastic extrusion is a continuous and complex process that integrates mixing, conveying, shearing, high temperature, pressure, and molding. The raw material undergoes complex structural and chemical changes during its passage through the extruder barrel until the processed material exits the extruder, which may or may not expand (Arora et al. 2020; Yi et al. 2022; Yu et al. 2023). Low‐moisture extrusion (moisture content below 35%) is typically employed to obtain more expanded and firm textured plant proteins, characteristics suitable for meat analogs such as burgers (Arora et al. 2020; Baune et al. 2022; McClements and Grossmann 2021b; Ye et al. 2009; Zhang et al. 2024).

There are several types of plant proteins that have demonstrated the necessary technical‐functional and nutritional attributes to substitute meat proteins. For example, soy and wheat proteins have been used to create analogs that mimic many of the desirable physicochemical, sensory, and nutritional attributes of animal protein (Bakhsh, Lee, Lee, Hwang, and Joo 2021; Ishaq et al. 2022; Mäkinen et al. 2017; Sultana et al. 2012). Soy is a readily available ingredient and is recognized for its excellent nutritional and functional attributes; it is abundant in carbohydrates, fats, fibers, vitamins, micro‐ and macronutrients (Bakhsh, Lee, Lee, Hwang, and Joo 2021; Ozturk and Hamaker 2023). There are also many other plant protein sources that may be used for this purpose, such as pea and rice, among others (Bakhsh, Lee, Lee, Sabikun, et al. 2021; Chiang et al. 2021; De Angelis et al. 2020; Lee et al. 2022). However, research focusing on combining soy protein concentrate, vital wheat gluten, and the inclusion of baru by‐product, particularly in meat analogs, remains scarce in the literature.

Among Brazil's native fruits, baru (*Dipteryx alata* Vog.) is valued primarily for its seed or almond (Alves‐Santos et al. 2021; de Miranda Monteiro et al. 2025). This seed is energy‐rich, with high levels of good‐quality proteins and lipids, dietary fiber, minerals, vitamins, and polyphenols. Its consumption contributes to improved lipid profile, body weight control, and oxidative stress mitigation (Alves‐Santos et al. 2021, 2023; Campos et al. 2024).

From pressing the baru seeds, a solid residue is obtained which is usually ground and converted into defatted baru flour, commonly used in preparing typical Cerrado dishes. However, there are reports that this by‐product is discarded in many situations (Aracava et al. 2022; Miranda et al. 2025). Research has shown, however, that this flour retains a significant amount of macro‐ and micronutrients from baru seeds (Miranda et al. 2025; de Aguiar et al. 2015), highlighting its great potential for the formulation of healthier foods, such as textured plant protein.

Despite this evidence and recognized potential, this by‐product remains underutilized (Alves‐Santos et al. 2023). Although baru is a native species, it is still little known and exploited in the current market. Valuing the use of by‐products like defatted baru flour could not only help reduce environmental impact but also represent a strategic opportunity for developing novel high‐value products, benefiting both local communities and the broader market (Alves‐Santos et al. 2021; Aracava et al. 2022).

In this context, the use of agroindustrial by‐products such as defatted baru flour (DBF) represents a promising approach for the production of textured vegetable proteins (TVPs), combining nutritional and environmental advantages. Therefore, this study aimed to evaluate the application of selected TVPs enriched with defatted baru flour, previously developed and characterized (Rebellato et al. 2025), in plant‐based burgers formulated with soy protein concentrate, defatted baru flour, and vital wheat gluten, focusing on their technological properties, nutritional composition, microbiological quality, and sensory acceptance.

## 2 Materials and Methods

### 2.1 Textured Vegetable Proteins

The textured vegetable proteins (TVPs) used in this study were obtained from a previous study (Rebellato et al. 2025). To ensure reproducibility, the key extrusion parameters are briefly described below. The blends (different proportions of defatted baru flour—DBF, soy protein concentrate—SPC, and vital wheat gluten—VWG) were subjected to the extrusion process using a co‐rotating twin‐screw extruder, ZSK 30 (Werner & Pfleiderer Corp., Ramsey, USA), with an L/D (length/diameter) ratio of 29:1. The extruder parameters were set as follows: feed rate of 7.5 kg/h, screw rotation speed of 300 rpm, heating zone temperatures of 60°C, 80°C, 135°C, and 135°C, and an outlet die with two 3 mm orifices.

The TVP formulations were defined based on the proportions of soy protein concentrate (SPC), defatted baru flour (DBF), and vital wheat gluten (VWG) in the dry ingredient blend. The values are expressed as percentages of the dry mixture (SPC:DBF:VWG), while conditioning moisture corresponds to the water added prior to extrusion. The formulations were as follows: E11 (control): 90:0:10 with 24% conditioning moisture; E1: 92:4:4 with 20.4% conditioning moisture; E3: 80:16:4 with 20.4% conditioning moisture; E9: 80:10:10 with 18% conditioning moisture; and E16: 80:10:10 with 24% conditioning moisture.

Although formulations E9 and E16 share the same ingredient ratio (80:10:10, SPC:DBF:VWG), they differ in conditioning moisture (18% and 24%, respectively), which was varied to evaluate the effect of moisture content during extrusion on the functional properties of the resulting TVPs.

### 2.2 Preparation of Plant‐Based Burgers

Before burger preparation, TVPs were hydrated with water at 4°C (1:10 w/w) for 1 h, and excess water was removed by manual pressing. The hydrated proteins were stored under refrigeration overnight.

Plant‐based burgers were prepared by mixing all ingredients (Table 1) in a planetary mixer (model FPSTSM2711, Oster, China) for 120 s at low speed. The resulting mixture was portioned (approximately 100 g) and molded into patties (112 mm diameter), which were packaged in plastic bags and frozen at −18°C for 48 h prior to analyses. For microbiological evaluation, samples were analyzed after 48 h of frozen storage (initial time) and after 60 days at −18°C to assess microbiological stability. A total of 25 replicates were performed for each treatment.

**TABLE 1:** Formulations (g/100 g) of plant‐based burgers.

Ingredient	Proportion (E: 11,1,3,9,16)
Hydrated textured vegetable protein	75
Vegetable fat	10
NaCl	1.0
Seasoning mix	0.8
Methylcellulose	1.5
Water	11.45

*Note:* Proportions of soy protein concentrate, defatted baru flour and vital wheat gluten used in TVPs: E11 (90:0:10, control); E1 (92:4:4); E3 (80:16:4); E9 (80:10:10); and E16 (80:10:10). E9 and E16 differ only in conditioning moisture. Vegetable fat: palm oil. Seasoning mix: garlic powder, monosodium glutamate, black pepper and cardamom.

For cooking, frozen burgers were grilled on a preheated griddle (model GR5, Cuisinart, USA) for approximately 6 min, being flipped every 3 min until reaching an internal temperature of 75°C, monitored using a digital thermometer (Delta OMH, model HD9218, Italy). After cooling to room temperature (30–40 min), the burgers were subjected to analysis. All physicochemical, microstructural, and sensory analyses were conducted on the cooked samples.

For sensory evaluation, samples were grilled immediately before testing following the procedure described above and kept in a heated oven to maintain temperature until evaluation.

### 2.3 Characterization of Plant‐Based Burgers

Moisture, protein, lipid, and ash contents were determined according to AACC methods 44–15.02, 46–13.01, 30–25.01, and 08–01.01, respectively (AACC 2010). Digestible carbohydrate content was calculated by difference.

Instrumental color was measured in the CIELab* system using a CM‐5 spectrophotometer (Konica Minolta, Tokyo, Japan) with D65 illumination, 10° observer angle, and SCE mode; four replicates were analyzed per batch. Sample pH was measured at 24°C–25°C using an F2‐Food pH meter (Mettler Toledo, USA) by direct insertion of the penetration probe (three independent replicates).

Cooking loss and shrinkage were calculated from weight and diameter differences before and after cooking, expressed as percentages (triplicate determinations).

Instrumental juiciness was evaluated following Lucherk et al. (2017), with modifications. Cubes (0.5 cm^3^) were weighed between two filter papers and compressed for 30 s at 78.45 N using a TA‐XT2i texture analyzer (Texture Technologies Corp., Scarsdale, USA) fitted with a 25 kg load cell and a 5 g trigger force. Samples were then reweighed and the exudate released was expressed as a percentage (four independent replicates).

Texture profile analysis (TPA) was performed at 25°C–27°C with the TA‐XT2i texture analyzer equipped with a 25 kg load cell and 5 g trigger force. Six independent samples (0.8 cm high, approx. 2.0 cm diameter) were compressed uniaxially to 50% of their original height in two consecutive cycles with a 2 s interval, using a cylindrical probe (Ø 50 mm) at a constant speed of 2 mm s^−1^. Hardness (N), springiness (mm), cohesiveness (dimensionless), and chewiness (N mm^−1^) were calculated.

For microstructural evaluation, burgers were freeze‐dried and examined under a TM 4000 scanning electron microscope (Hitachi Technologies, Tokyo, Japan) at 10 kV accelerating voltage (mode 4), 52,000 nA emission current, vacuum level 50 (Chg‐up Red. L), and BSE imaging mode with shadow 2 configuration.

### 2.4 Microbiological Quality Assessment

Microbiological tests were carried out on frozen samples both initially (48 h) and after 60 days of frozen storage at −18°C to detect *Salmonella* and to enumerate coagulase‐positive *Staphylococci*, molds, yeasts, psychrotrophic bacteria, and thermotolerant coliforms, following the procedures described by Salfinger and Tortorello (2015).

### 2.5 Sensory Analysis

Sensory evaluation was conducted with 87 volunteers. All participants signed an informed consent form approved by the Research Ethics Committee of the University of Campinas (approval no. 6.086.010, CAAE 67076023.2.0000.5404) before testing. Each participant evaluated four burger samples: one control (E11) prepared with soy protein and vital wheat gluten, and three experimental burgers formulated with TVPs containing soy protein concentrate, defatted baru flour and vital wheat gluten (E1, E3, E16). Notably, formulation E9 (18% moisture) was not included in the sensory evaluation because of its fragile and brittle texture, which also made the measurement of texture parameters unfeasible. Three sensory tests were performed: (i) acceptance of appearance, color, aroma, flavor, texture and overall impression; (ii) purchase intention; and (iii) Check‐All‐That‐Apply (CATA) analysis.

Samples were served on disposable plates coded with random three‐digit numbers and presented in a balanced order. Acceptance of color, aroma, flavor, texture and overall impression was scored using a nine‐point hedonic scale (1 = dislike extremely; 9 = like extremely). The CATA test used a list of 18 descriptive attributes from which participants selected all terms applicable to each sample (Ares et al. 2010). Purchase intention was recorded on a five‐point structured scale (1 = certainly would not buy; 5 = certainly would buy).

### 2.6 Statistical Analysis

Data were screened for outliers and tested for normality (Shapiro–Wilk test) at a 95% confidence level (*p* < 0.05). One‐way analysis of variance (ANOVA) followed by Tukey's test was applied to identify significant differences among treatments at a 95% confidence level (*p* < 0.05). All analyses were performed using IBM SPSS Statistics version 20. For sensory evaluation, acceptance and purchase intention data were analyzed using ANOVA and Tukey's test (*p* < 0.05). CATA data were analyzed by calculating the frequency of mention for each descriptor, applying a non‐parametric Cochran's Q test to identify significant differences among samples. Correspondence analysis was used to visualize the relationship between samples and sensory descriptors, and principal component analysis (PCA) was applied to explore associations between CATA attributes and overall liking. These analyses were performed using XLSTAT 2018 (Addinsoft, New York, USA).

## 3 Results and Discussion

### 3.1 Characterization of Plant‐Based Burgers

Characterization data for the grilled burgers formulated with soy‐based textured protein, baru by‐product, and vital wheat gluten are summarized in Table 2. Both proximate composition and the other evaluated parameters varied significantly as a function of the TVP composition used.

**TABLE 2:** Characterization of plant‐based burgers.

Parameters	Treatments				
	E11 control	E1	E3	E9	E16
**Proximate composition (%)**					
Moisture	62.10 ± 0.43a	58.53 ± 0.58b	58.53 ± 0.31b	58.12 ± 0.38b	57.71 ± 0.17b
Proteins	25.82 ± 0.40b	27.61 ± 0.02a	17.59 ± 0.70c	26.62 ± 0.65ab	27.85 ± 0.72a
Lipids	9.17 ± 0.25b	9.91 ± 0.16ab	10.87 ± 0.35a	10.93 ± 0.52a	10.48 ± 0.62a
Ash	2.48 ± 0.03a	2.39 ± 0.08ab	2.39 ± 0.06ab	2.24 ± 0.15b	2.35 ± 0.04ab
Carbohydrates	0.43	1.56	10.62	2.09	1.61
**pH**	6.32 ± 0.02c	6.69 ± 0.02b	6.68 ± 0.01b	6.70 ± 0.02b	6.80 ± 0.01a
**Instrumental color**					
L*	52.10 ± 1.50a	48.94 ± 1.00b	45.59 ± 0.90d	46.81 ± 1.30 cd	47.57 ± 1.10bc
a*	8.11 ± 0.50a	4.96 ± 0.50d	6.14 ± 0.30b	5.46 ± 0.50c	5.91 ± 0.20bc
b*	21.47 ± 0.90a	12.82 ± 1.20b	12.75 ± 0.80b	11.98 ± 0.90b	12.84 ± 0.50b
**Technological characteristics**					
Cooking yield (%)	91.22 ± 1.60a	85.08 ± 2.00c	84.13 ± 0.74 cd	88.17 ± 1.73b	82.47 ± 1.09d
Shrinkage (%)	3.13 ± 0.86ab	2.88 ± 0.72b	4.13 ± 0.88a	3.50 ± 0.54ab	3.59 ± 0.33ab
Instrumental juiciness (%)	6.24 ± 0.81a	4.61 ± 0.57b	4.85 ± 0.79b	4.44 ± 0.57b	4.49 ± 0.70b
**Textural parameter**					
Hardness (N)	9.31 ± 1.20c	10.53 ± 1.90b	16.94 ± 1.60a		15.84 ± 2.10a
Springiness (mm)	0.58 ± 0.06a	0.57 ± 0.10a	0.43 ± 0.03b	—	0.46 ± 0.03b
Cohesiveness	0.38 ± 0.03a	0.35 ± 0.04b	0.29 ± 0.01c	—	0.29 ± 0.01c
Resilience	0.16 ± 0.02a	0.16 ± 0.03a	0.09 ± 0.01b	—	0.09 ± 0.01b

*Note:* Values are mean ± SD (*n* = 3). Within a row, means with the same letter do not differ significantly (*p* > 0.05; Tukey's test). Treatments: E11 (90:0:10, control), E1 (92:4:4), E3 (80:16:4), E9 (80:10:10), and E16 (80:10:10). Proportions refer to SPC:DBF:VWG in the TVP used to prepare the plant‐based burgers. E9 and E16 differ only in conditioning moisture.

The control burger, prepared with soy protein concentrate and vital wheat gluten, showed the highest moisture content and the lowest digestible carbohydrate content compared with formulations containing baru by‐product.

With respect to protein content, treatments E1 and E16 (24% moisture) exhibited higher protein levels than the control, which was formulated solely with soy protein concentrate and vital wheat gluten. This finding is particularly relevant, as it shows that adding defatted baru flour at low levels (4%–10%) contributed positively to the protein content of the formulations. By contrast, only sample E3 showed a lower value (17.6%) than the other burgers (≈25%); however, its carbohydrate content was higher. This outcome is likely related to the composition of the TVP used in that treatment, which contained a lower proportion of soy concentrate and a higher proportion of defatted baru flour. Although protein content was reduced, the higher carbohydrate content may provide more energy and dietary fiber, which support health by helping prevent chronic diseases and increasing satiety (Miranda et al. 2025).

The higher lipid content observed in burgers containing baru by‐product is attributable to the greater residual fat in defatted baru flour (≈7%) compared with soy protein concentrate (0.2%) (Rebellato et al. 2025). Baru almonds contain approximately 50% monounsaturated and 30% polyunsaturated fatty acids, with high levels of oleic acid (C18:1) and linoleic acid (C18:2ω6), respectively (Alves‐Santos et al. 2021). Such a profile is considered beneficial to health, as diets rich in unsaturated fatty acids can reduce risk factors, as well as morbidity and mortality associated with cardiovascular disease (Billingsley et al. 2018).

Alves‐Santos et al. (2021) conducted a comprehensive review of baru (*Dipteryx alata* Vog.), noting that almond composition is quite variable: protein ranges from 20% to 31%, lipids from 24% to 46%, ash from 1.6% to 3.3%, and carbohydrates from 9% to 23%, of which 6%–14% are total fiber (0.9%–2.5% soluble; 7%–13% insoluble). Few studies have assessed the composition of baru by‐products. de Oliveira Pineli et al. (2015) reported that partially defatted baru flour obtained after oil extraction contains 29.50% protein, 11.80% lipids, 11.60% carbohydrates, and 38.80% total fiber (5.07% soluble; 33.70% insoluble), underscoring its macronutrient contribution.

Compared with values reported by Luz et al. (2024) for commercial plant‐based burgers produced in Brazil, formulated with various cereals, legumes, vegetables, seeds, fruits, mushrooms, roots, and tubers (e.g., soybeans, peas, chickpeas, lentils, beans, corn, cassava, potatoes, oats, quinoa, chia, green jackfruit, and shiitake), the burgers developed in the present study using TVPs containing baru by‐product exhibited higher protein and lipid contents and lower carbohydrate levels. On average, the commercial products reported by Luz et al. (2024) contained 11% protein, 5% lipids, and 18% carbohydrates. Relative to Cutroneo et al. (2023), who compared plant‐based and beef burgers, the present burgers had higher protein and lower lipid contents. These results indicate that the TVPs with baru by‐product can yield plant‐based burgers with protein contents superior to the commercial products analyzed in those studies.

The pH of the plant‐based burgers ranged from 6.3 to 6.8 and was higher (*p* < 0.05) in samples containing baru by‐product. According to Alves‐Santos et al. (2021), baru almonds are rich in arginine, a basic amino acid, which may explain the higher pH values in the baru‐containing burgers. pH values close to 6 have been reported for plant‐based burgers (textured soy, peanut flour and pea fiber), with variability attributed to ingredient alkalinity/acidity and overall formulation diversity (Botella‐Martínez et al. 2022).

**FIGURE 1:**
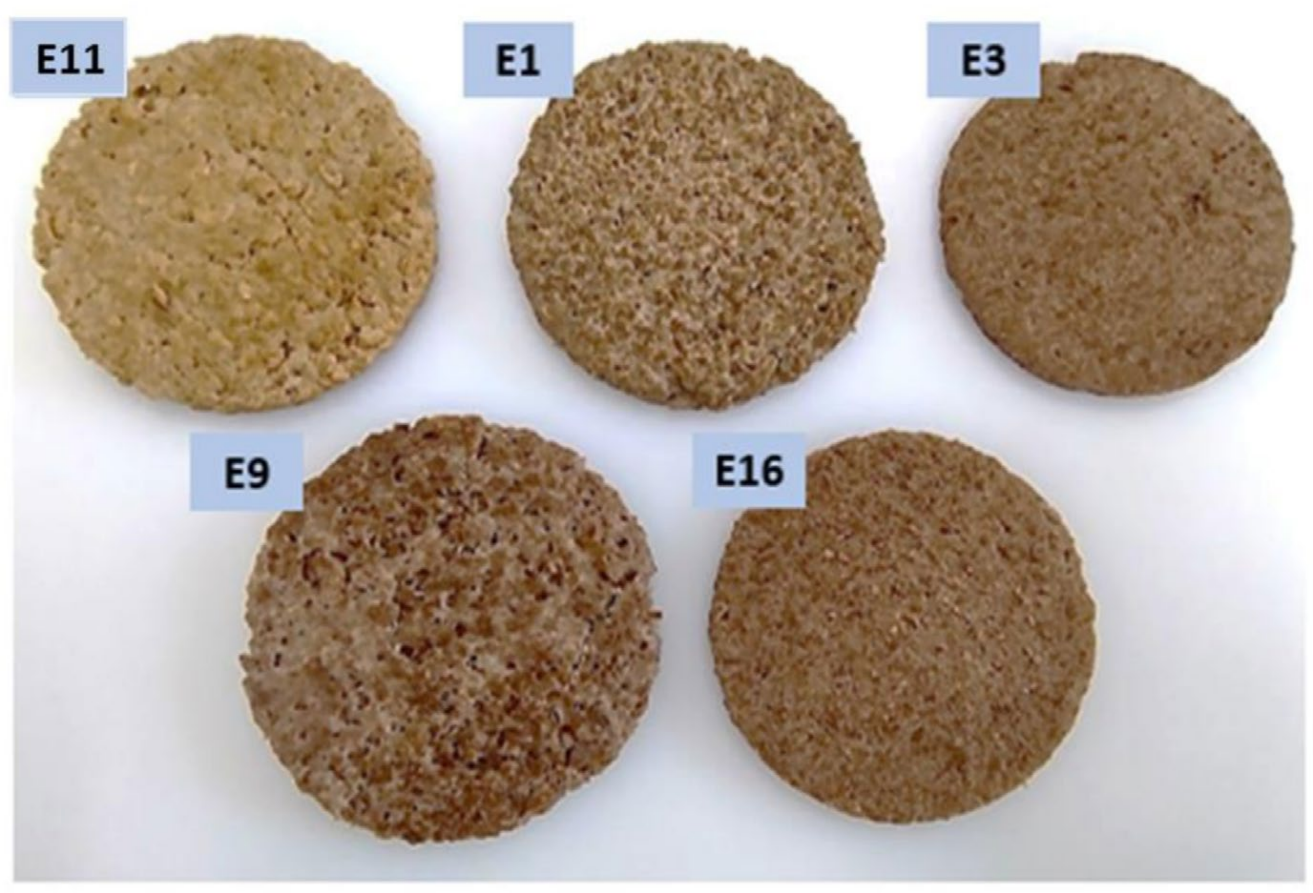
Visual characteristics of grilled plant‐based burgers. E11: 90:0:10 (control); E1: 92:4:4; E3: 80:16:4; E9: 80:10:10; E16: 80:10:10 (SPC:DBF:VWG in TVP). E9 and E16 differ only in conditioning moisture.

Regarding color, the control burger exhibited higher L* (52.10), a* (8.11) and b* (21.47) values, differing significantly from the baru formulations, which appeared darker with lower intensities of red (a*) and yellow (b*) tones (Figure 1). This outcome relates to the inherent color of the raw materials used to produce the TVPs. Defatted baru flour is characteristically brown and rich in phenolics and reducing sugars, favoring non‐enzymatic browning during extrusion and cooking (Alves‐Santos et al. 2021; de Oliveira Pineli et al. 2015; Rebellato et al. 2025). Compared with soy protein concentrate and vital wheat gluten, baru flour has lower lightness (L*), lower yellowness (b*), and higher redness (a*) (Rebellato et al. 2025). Consequently, the decrease in L* was most pronounced in sample E3, which had the lowest value (45.59), evidencing the strong influence of baru flour level on final color.

In developing meat analogs such as plant‐based burgers, consumer acceptance is strongly linked to visual appearance. After achieving appropriate texture and shape, color and its changes during preparation and cooking become key targets. Because the main ingredients used in plant‐based burgers (soy and wheat proteins) are predominantly beige/yellowish (Botella‐Martínez et al. 2022; Kyriakopoulou et al. 2021), incorporating defatted baru flour contributed positively to a reddish tone typical of traditional burgers. Ideally, color should be stable at the product's pH and undergo desirable changes during cooking, such as browning (Botella‐Martínez et al. 2022; Kyriakopoulou et al. 2021). In this context, TVP combined with defatted baru flour proved promising as it yielded a darker appearance after cooking, resembling typical browning observed in thermally processed products.

For technological traits, the control burger (E11, with no baru by‐product in the TVP) differed significantly (*p* < 0.05) from the baru treatments. The control showed the highest cooking yield (91.22%), indicating greater mass retention after cooking, whereas E16 (24% moisture) had the lowest yield (82.47%). These data suggest that TVP without baru (E11) promoted greater water and/or fat retention, characteristics commonly associated with improved technological performance in meat analogs. Nevertheless, E9 (18% moisture) achieved a cooking yield (88.17%) similar to the control, indicating good performance (Table 2).

Shrinkage did not differ significantly (*p* > 0.05) between the control and the baru‐containing burgers, which is positive and suggests that TVPs based on SPC and VWG—already well accepted by consumers—can be complemented with baru by‐product without compromising this attribute.

For instrumental juiciness, the control burger released the highest exudate (6.24%), differing from the baru treatments (*p* < 0.05). Instrumental juiciness was quantified as liquid released during compression, simulating mastication; higher values indicate greater juiciness. By contrast, baru‐containing burgers released less liquid, suggesting greater retention upon compression, which may be associated with a firmer structure and potentially lower juiciness perception.

Texture properties differed significantly (*p* < 0.05) among treatments for hardness, springiness, cohesiveness, and resilience. Notably, texture parameters could not be measured for E9 (18% moisture) due to brittleness, which also precluded its subsequent sensory evaluation. Overall, adding baru by‐product to the TVPs increased hardness and decreased springiness, cohesiveness, and resilience relative to the control. Greater firmness may be associated with higher fiber contents in E3 and E16 (24% moisture), contributing to increased mechanical resistance. In addition to fiber, interactions among plant proteins, fiber, and starch present in the ingredients should also be considered as relevant factors influencing the mechanical properties of plant‐based burgers (Botella‐Martínez et al. 2022). Texture was the attribute that differentiated the burgers sensorially: the control (E11) had the lowest mean score (5.09), significantly lower than the others, especially E16 (24% moisture) (6.37), which had the best acceptance, suggesting that the incorporation of baru by‐product contributed to greater texture acceptance, possibly related to changes in structure and moisture retention.

Firmness increases in DBF‐containing burgers are consistent with a denser, less elastic network. The increase in firmness observed in DBF‐containing burgers may be related to the presence of insoluble fiber and residual lipids, which can influence the structuring of the TVP protein matrix. However, the mechanisms underlying these effects were not directly evaluated in this study.

### 3.2 Microstructure of Textured Vegetable Proteins and Plant‐Based Burgers

**FIGURE 2:**
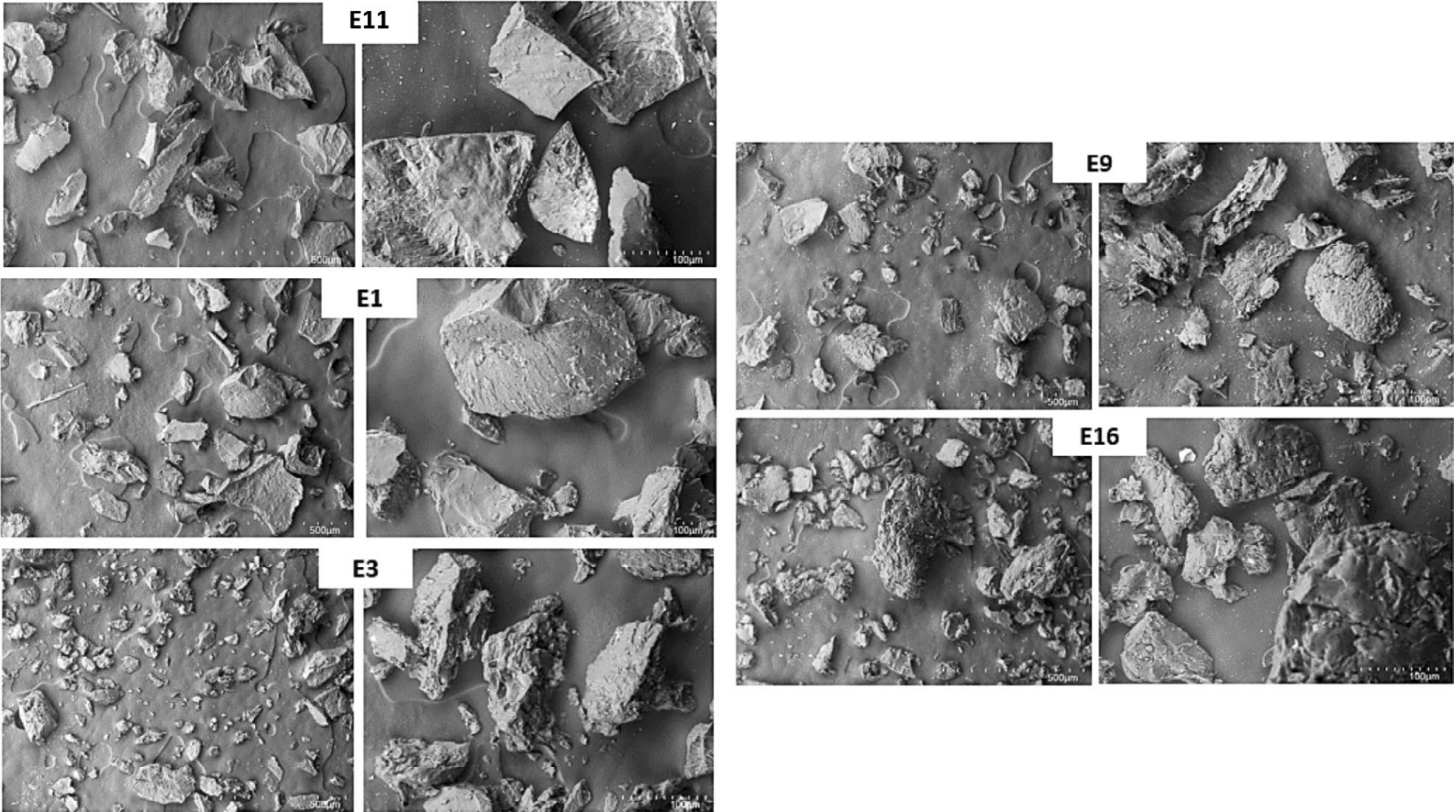
Microstructure of textured proteins at 100× and 300×. E11: 90:0:10 (control); E1: 92:4:4; E3: 80:16:4; E9: 80:10:10; E16: 80:10:10 (SPC:DBF:VWG in TVP). E9 and E16 differ only in conditioning moisture.

Morphological analysis revealed significant changes in the surface microstructure of particles as a function of TVP composition. The control (E11) showed larger particles with flat surfaces and well‐defined fractures. Due to the low level of defatted baru flour, E1 presented features similar to the control (Figure 2).

As the concentration of defatted baru flour increased (E3 to E16), progressive particle fragmentation was observed, together with greater porosity and morphological irregularity. E9 (18% moisture) and E16 (24% moisture) (both 10% baru flour) exhibited more disorganized structures, with rough surfaces and highly deformed morphologies, possibly due to interference of the flour with protein matrix formation during extrusion. E3, the formulation with the highest baru flour level, displayed a particularly distinct morphology characterized by numerous smaller particles and a markedly more porous surface. These changes may be linked to the flour's fiber content, which could promote greater matrix disruption and cell rupture during extrusion.

**FIGURE 3:**
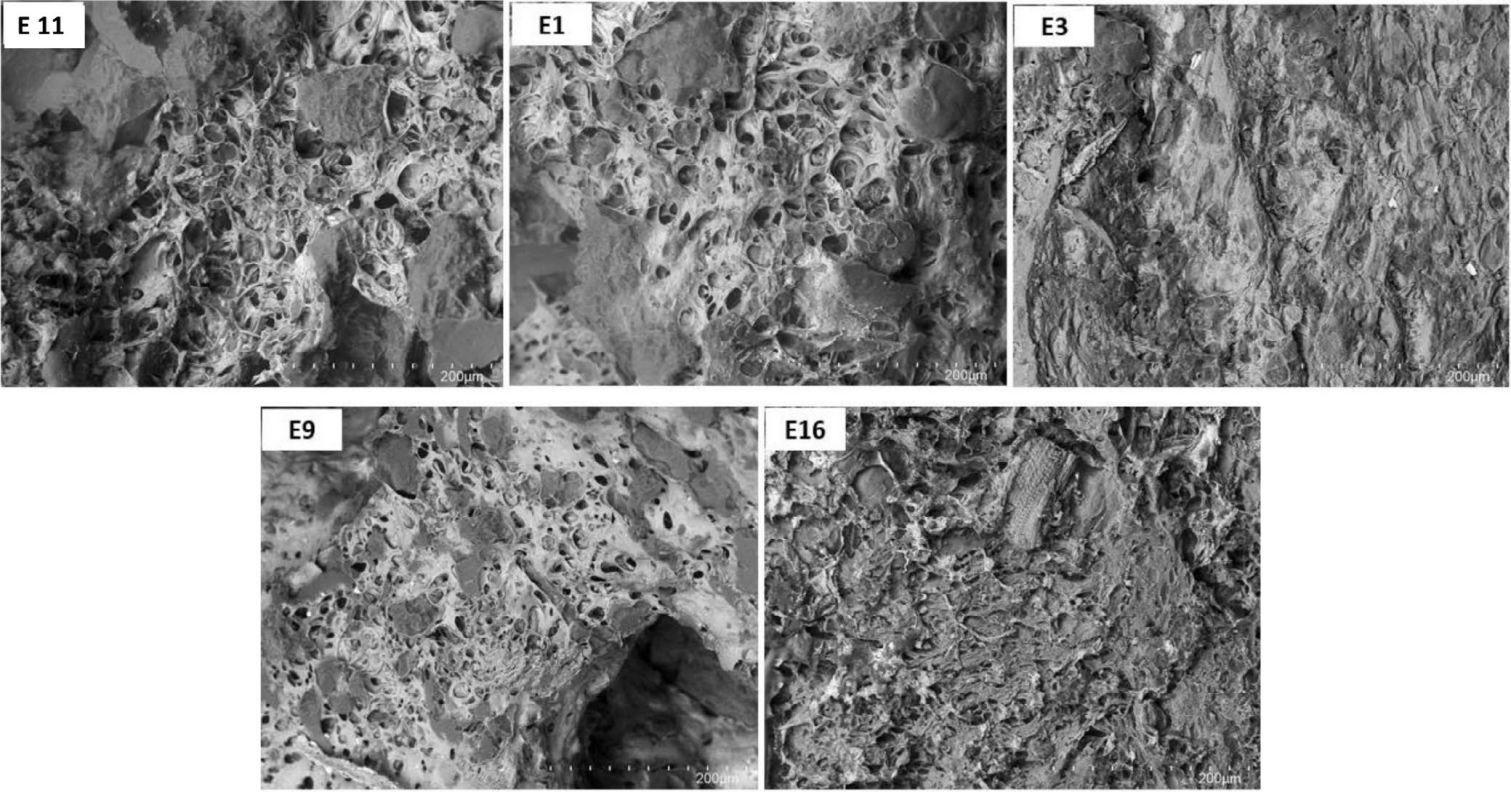
Microstructure of plant‐based burgers at 250×. E11: 90:0:10 (control); E1: 92:4:4; E3: 80:16:4; E9: 80:10:10; E16: 80:10:10 (SPC:DBF:VWG in TVP). E9 and E16 differ only in conditioning moisture.

Figure 3 shows the internal structures of burgers formulated with different ingredient proportions. The control (E11) exhibited a well‐defined protein network with a porous structure. A similar pattern was observed in E1 and E9 (18% moisture), albeit with differences in porosity. In contrast, E3 and E16 (24% moisture) did not present the characteristic protein network.

The images indicate that adding defatted baru flour induces relevant microstructural changes. Control (E11) and E1 (lower baru level) showed more porous, aerated structures with well‐defined cavities, indicative of a less dense matrix and potentially associated with a softer texture. With higher baru proportion—10% (E9 and E16, 18% and 24% moisture, respectively), porosity decreased and matrix compaction increased. E3 (highest baru level) showed a dense, low‐porosity microstructure, suggesting greater component aggregation and reduced matrix expansion, which may compromise desirable sensory attributes such as juiciness and tenderness, resulting in a firmer texture and more resistant mastication.

E1 (92% soy protein, 4% baru flour, 4% gluten) showed alterations in the protein network, likely related to the presence of baru flour, despite its higher soy protein level compared with the control. E3, with the highest baru level, did not form the characteristic protein network, probably due to its composition, which, consistent with Table 2, had the lowest protein and highest carbohydrate contents among the formulations. In E9 (18% moisture), a less developed protein network with lower porosity was observed than in the control and E1, both richer in soy protein and poorer in baru flour. Although E9 (18% moisture) and E16 (24% moisture) shared the same SPC:DBF:VWG ratio, E16 was processed at higher feed moisture, which typically reduces melt viscosity and shear‐driven alignment during extrusion. The resulting matrix is less fibrillar and more compact, consistent with the weaker protein network seen in E16.

Overall, reduced protein networking resulted in denser burgers and, consequently, greater hardness, as shown in Table 2. Similar findings were reported by Palanisamy et al. (2018) when evaluating soy‐based plant burgers with added carrageenan, where progressive network reduction occurred as carbohydrate level increased.

### 3.3 Microbiological Quality of Plant‐Based Burgers

Prior to sensory analysis, samples were assessed for microbiological quality (Table 3). Although counts varied slightly over storage, psychrotrophic bacteria and mold counts were similar at the initial time and after 60 days of freezing. *Salmonella* was not detected in any sample. Counts of coagulase‐positive staphylococci (*m* ≤ 5 × 10^2^ CFU g^−1^) and molds and yeasts (*m* ≤ 10^2^ CFU g^−1^) remained within satisfactory limits (m), indicating that the burgers were suitable for consumption. Applicable limits for each microorganism are specified in Brazil's Normative Instruction No. 161/2022 (Brasil 2022).

**TABLE 3:** Microbiological quality of plant‐based burgers during frozen storage.

Microorganisms	E11 control		E1		E3		E9		E16	
	T1	T2	T1	T2	T1	T2	T1	T2	T1	T2
*Salmonella* ^a^	Abs	Abs	Abs	Abs	Abs	Abs	Abs	Abs	Abs	Abs
Staphylococcus coagulase‐positive^b^	< 10^2^	< 10^2^	< 10^2^	< 10^2^	< 10^2^	< 10^2^	< 10^2^	< 10^2^	< 10^2^	< 10^2^
Molds^b^	1.0 × 10^3^	< 10^2^	< 10^2^	< 10^2^	< 10^2^	< 10^2^	< 10^2^	< 10^2^	< 10^2^	< 10^2^
Yeast^b^	< 10^2^	< 10^2^	< 10^2^	< 10^2^	< 10^2^	< 10^2^	< 10^2^	< 10^2^	< 10^2^	< 10^2^
Psicotróficos^b^	< 10^2^	< 10^2^	< 10^2^	2.0 × 10^2^	< 10^2^	< 10^2^	< 10^2^	1.0 × 10^2^	< 10^2^	1.0 × 10^2^
Coliforms termotolerantes^c^	< 3.0	< 3.0	< 3.0	< 3.0	< 3.0	< 3.0	< 3.0	< 3.0	< 3.0	< 3.0

*Note:* Abs = Absent: not detected. T1: baseline (immediately after freezing, 48 h). T2: 60 days frozen. Treatments: E11 (90:0:10, control), E1 (92:4:4), E3 (80:16:4), E9 (80:10:10), and E16 (80:10:10). Proportions refer to SPC:DBF:VWG in the TVP used to prepare the plant‐based burgers. E9 and E16 differ only in conditioning moisture.

^a^ Detection of *Salmonella* in 25 g of sample.

^b^ CFU g^−1^: colony‐forming units per gram.

^c^ MPN g^−1^: most probable number per gram.

Additionally, although there is no specific Brazilian standard for psychrotrophic counts in semi‐prepared foods, good manufacturing practice suggests that counts below 10^6^ CFU g^−1^ are recommended for refrigerated products. For thermotolerant coliforms in unspecified semi‐prepared foods, counts up to 100 CFU g^−1^ are considered satisfactory (Marioto et al. 2020).

### 3.4 Sensory Properties of Plant‐Based Burgers

Control (E11), E1, E3 and E16 (24% moisture) burgers were submitted to sensory testing to gain deeper insight into sensory characteristics and consumer perceptions considering beliefs, dietary habits and consumption of meat analogs. The panel consisted of 87 participants, 52 female (60%) and 35 male (40%). Regarding age distribution, 56 participants (64%) were 18–25 years old, 26 (30%) were 26–35 years old, and 5 (6%) were 36–45 years old. Most participants identified themselves as meat consumers (63; 72%), followed by flexitarians (17; 19%), vegetarians (6; 7%), and vegans (2; 2%).

Previous experience with plant‐based burgers was reported as frequent by 6 participants (7%), occasional by 49 (56%), and absent for 32 (37%). Percentages are calculated over the total sample; totals may not equal 100% due to rounding.

Sensory results indicate that the inclusion of baru by‐product contributed positively to several attributes compared with the control (E11), as shown in Table 4. For appearance and color, all baru formulations (E1, E3, E16) achieved significantly higher mean scores (*p* < 0.05) than the control, likely due to the more intense color imparted by defatted baru flour, which improved visual appeal.

**TABLE 4:** Mean acceptance scores^a^ and purchase intention^b^ for plant‐based burgers made with soy TVP, baru by‐product, and vital wheat gluten.

Sensory attributes	Treatments			
	E11 control	E1	E3	E16
Appearance	6.34 ± 1.85b	7.75 ± 1.21a	7.16 ± 1.53a	7.39 ± 1.43a
Color	6.05 ± 2.08b	7.75 ± 1.38a	7.21 ± 1.56a	7.53 ± 1.36a
Aroma	6.70 ± 1.73a	7.16 ± 1.66a	7.17 ± 1.75a	6.97 ± 1.46a
Flavor	6.02 ± 1.83a	6.48 ± 1.88a	6.44 ± 1.58a	6.59 ± 1.66a
Texture	5.09 ± 1.91c	6.22 ± 2.10ab	5.52 ± 1.84bc	6.37 ± 1.84a
Overall impression	5.78 ± 1.60b	6.63 ± 1.82a	6.45 ± 1.45a	6.75 ± 1.51a
Purchase intention	2.47 ± 1.03b	3.14 ± 1.22a	2.91 ± 1.08a	3.23 ± 1.06a

*Note:* Identical letters within a row indicate no significant differences (*p* > 0.05; Tukey's test). E11: 90:0:10 (control); E1: 92:4:4; E3: 80:16:4; E16: 80:10:10 (SPC:DBF:VWG in TVP). E9 and E16 differ only in conditioning moisture.

^a^ Nine‐point hedonic scale (1 = dislike extremely; 9 = like extremely).

^b^ Five‐point scale (1 = certainly would not buy; 5 = certainly would buy).

No significant differences were observed among samples for aroma and flavor, indicating that baru by‐product did not impair olfactory or gustatory acceptability, suggesting successful integration into the sensory matrix without negative impacts on organoleptic characteristics.

Texture most clearly differentiated the burgers. The control (E11) had the lowest mean (5.09), significantly lower than the others, particularly E16 (6.37), which had the best acceptance for texture. This suggests that baru by‐product may have contributed to a more pleasant texture, possibly by influencing the protein matrix and moisture retention.

Overall impression and purchase intention followed the same trend: baru burgers (E1, E3, E16) showed the highest means and were significantly superior (*p* < 0.05) to the control. Thus, the addition of baru by‐product improved color, appearance, and texture, as well as overall liking and purchase intention, underscoring the technological and sensory potential of incorporating baru by‐products into TVPs for plant‐based burgers.

**FIGURE 4:**
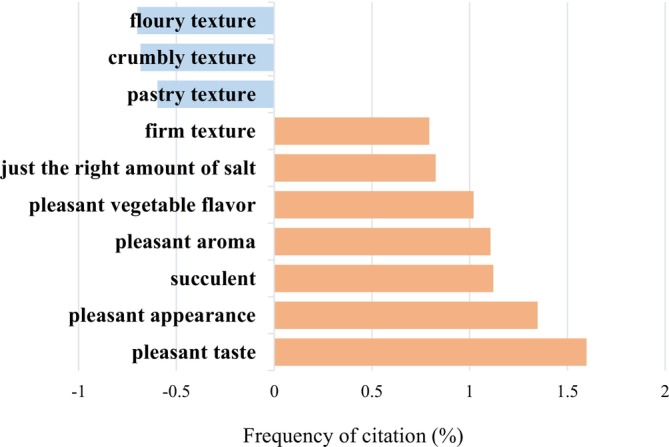
Attributes influencing sensory acceptance of plant‐based burgers formulated with soy TVP, baru by‐product, and vital wheat gluten.

Figure 4 summarizes the most frequently cited positive and negative sensory attributes for the burgers formulated with TVPs containing soy protein concentrate, baru by‐product, and vital wheat gluten.

Positively cited attributes included pleasant flavor, appearance, aroma and vegetal flavor; salt level was judged adequate. Juiciness and firm texture were also viewed positively. Conversely, texture was also a negative driver for some participants, being described as pasty, crumbly and floury. Pleasant flavor was the most valued attribute, which is noteworthy because soy‐based burgers often show residual or bitter notes inherent to soy composition (Botella‐Martínez et al. 2022). Attractive appearance strongly reinforced positive expectations, suggesting that color and shape were appealing. Plant‐based analogs often appear opaque and lack the red/pink hues characteristic of meat, making color replication difficult (Su et al. 2024). Frequent mentions of juiciness and pleasant aroma suggest that moisture release and volatile compounds generated during cooking were well received. The vegetal flavor, often a source of rejection, was perceived positively here. Unpleasant notes such as bitterness, beany and astringent flavors commonly hinder consumer preference for plant alternatives (Variyar and Mishra 2024).

Adequate salting and firm texture contributed to overall balance, indicating seasoning harmony and a structure that does not disintegrate easily. Nevertheless, negative texture perceptions persisted in some cases: a pasty texture suggests excess moisture, low cohesion or poor ingredient interaction; a crumbly texture may indicate insufficient protein binding, low moisture retention, or excess fiber; and a floury texture may reflect dryness or insufficient hydration/integration of baru flour with the protein matrix. Fundamental differences between plant and animal proteins in composition and structure contribute to a substantial gap between plant‐based and meat textures (Su et al. 2024).

Overall, burgers were well evaluated for flavor, appearance, juiciness and aroma, though challenges remain for texture and may be addressed through formulation or process adjustments, for example, moisture tuning, texturizing ratios or binders, to optimize texture and overall sensory acceptance.

**FIGURE 5:**
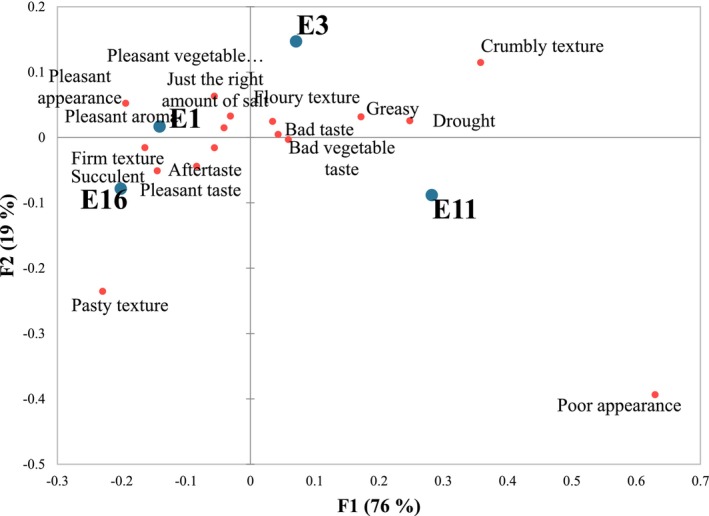
Check‐All‐That‐Apply (*n* = 87) for plant‐based burgers made with soy and baru TVPs. E11: 90:0:10 (control); E1: 92:4:4; E3: 80:16:4; E16: 80:10:10 (SPC:DBF:VWG in TVP).

Figure 5 presents the Check‐All‐That‐Apply (CATA) biplot, where axis F1 explains 76% of the variance and F2 explains 19% (95% total), indicating a highly representative distribution of samples and attributes. The control (E11), located in the lower‐right quadrant, was associated with negative attributes such as “unpleasant vegetal flavor,” indicating inferior sensory perception and reinforcing the limited role of the base formulation (soy concentrate + gluten, without baru) in sensory quality. Soy or pea protein isolates are the primary raw materials in plant‐based meat analogs (Bohrer 2019); however, characteristic compounds of cereals/legumes, especially in soy, can negatively impact flavor and acceptance (Kyriakopoulou et al. 2021).

E16 (24% moisture) (lower‐left quadrant) was associated with positive attributes such as firm texture, juiciness and pleasant flavor, suggesting that baru addition in this formulation contributed to a significant sensory improvement. E1 was associated with pleasant aroma and appearance, pleasant vegetal flavor, and adequate salt, indicating good sensory balance, especially for aroma and appearance. E3 (upper‐right quadrant) was not strongly associated with either highly positive or negative attributes; its relatively isolated position suggests an intermediate profile.

Negative attribute, pasty texture, poor appearance, crumbly texture and unpleasant flavor were clearly distant from E1 and E16 (24% moisture), reinforcing their superior sensory performance compared with the control (E11) and E3. Overall, the CATA results show that including baru by‐product at 4%–10% contributed positively to sensory quality. E1 and E16 were associated with desirable attributes such as pleasant flavor, juiciness and good appearance, whereas the control and E3 were linked to less desirable attributes, evidencing the technological and sensory potential of incorporating < 16% baru by‐product into the formulation.

Despite advances in plant‐based foods, sensory studies indicate that animal protein still enjoys higher acceptance because replicating meat's texture, flavor, color, and complex Maillard‐derived attributes remains challenging for plant‐based analogs (da Silva et al. 2025). The sector faces a major technological dilemma: pursue close mimicry of animal‐protein sensory experience, still difficult despite progress, or embrace a distinct sensory profile and risk lower acceptance among consumers seeking familiarity (Hernandez et al. 2023; Sendhil et al. 2024).

## 4 Conclusion

Incorporating defatted baru flour (DBF) into textured vegetable proteins based on soy and vital wheat gluten enabled the development of high‐protein plant‐based burgers with darker color after cooking and improved consumer acceptance compared to the control. Formulations containing 4% to 10% DBF in the TVP (E1 and E16) showed protein contents comparable to or higher than the control, satisfactory microbiological quality after 60 days of frozen storage, and significantly higher scores for color/appearance, texture, overall liking and purchase intention. Microstructural observations supported the sensory outcomes: moderate DBF levels preserved a porous protein network, whereas higher DBF and/or increased extrusion moisture promoted matrix densification, greater hardness and reduced springiness/cohesiveness; and, in one case (E9, 18% moisture), brittleness that impaired sensory assessment. Overall, the results suggest that DBF can be incorporated within a specific range to balance nutritional composition and sensory properties in plant‐based burgers. However, the mechanisms underlying the structural changes were not directly investigated and warrant further study. The use of DBF also represents a promising strategy for the valorization of regional by‐products, contributing to more sustainable food systems. Future research should focus on further refining DBF levels, optimizing extrusion moisture, and exploring innovative binder systems to mitigate excessive firmness in certain formulations, while continuously maintaining desirable color and protein enrichment.

## Author Contributions

**Ana Paula Rebellato:** conceptualization, data curation, formal analysis, methodology, validation, investigation, writing – original draft, writing – review and editing, funding acquisition, resources, supervision. **Mirian dos Santos:** data curation, formal analysis, methodology, validation, writing – review and editing. **Victor Guilherme Sebastião:** data curation, methodology, formal analysis, validation, writing – review and editing. **Caroline Joy Steel:** conceptualization, project administration, writing – review and editing, resources, supervision. **Joyce Grazielle Siqueira Stürmlinger:** conceptualization, writing – review and editing, project administration. **Marise Aparecida Rodrigues Pollonio:** conceptualization, writing – review and editing. **Bruna dos Reis Gasparetto Cruz:** data curation, formal analysis, methodology, validation, writing – review and editing.

## Funding

This work was supported, in whole or in part, by The Good Food Institute (GFI). The Coordination for the Improvement of Higher Education Personnel (CAPES), the National Council for Scientific and Technological Development (CNPq).

## Ethics Statement

This study involved human participants for sensory evaluation and was approved by the Research Ethics Committee of the Universidade Estadual de Campinas (approval no. 6.086.010, CAAE 67076023.2.0000.5404).

## Consent

Informed consent was obtained from all participants prior to their inclusion in the study.

## Conflicts of Interest

The authors declare no conflicts of interest.

## Data Availability

The datasets generated and/or analyzed during the current study are available from the corresponding author on reasonable request.
